# Consumption Optimization in an Office Building Considering Flexible Loads and User Comfort

**DOI:** 10.3390/s20030593

**Published:** 2020-01-21

**Authors:** Mahsa Khorram, Pedro Faria, Omid Abrishambaf, Zita Vale

**Affiliations:** 1 GECAD—Research Group on Intelligent Engineering and Computing for Advanced Innovation and Development, Rua DR. Antonio Bernardino de Almeida, 431, 4200-072 Porto, Portugal; makgh@isep.ipp.pt (M.K.); ombaf@isep.ipp.pt (O.A.); 2 Polytechnic of Porto, Rua DR. Antonio Bernardino de Almeida, 431, 4200-072 Porto, Portugal; zav@isep.ipp.pt

**Keywords:** building energy management, demand response, load shifting, SCADA, user comfort

## Abstract

This paper presents a multiperiod optimization algorithm that is implemented in a Supervisory Control and Data Acquisition system. The algorithm controls lights and air conditioners as flexible loads to reduce the consumption and controls a dishwasher as a deferrable load to implement the load shifting. Several parameters are considered to implement the algorithm for several successive periods in a real building operation. In the proposed methodology, optimization is done regarding user comfort, which is modeled in the objective function related to the indoor temperature in each room, and in the constraints in order to prevent excessive power reduction, according to users’ preferences. Additionally, the operation cycle of a dishwasher is included, and the algorithm selects the best starting point based on the appliance weights and power availability in each period. With the proposed methodology, the building energy manager can specify the moments when the optimization is run with consideration of the operational constraints. Accordingly, the main contribution of the paper is to provide and integrate a methodology to minimize the difference between the actual and the desired temperature in each room, as a measure of comfort, respecting constraints that can be easily bounded by building users and manager. The case study considers the real consumption data of an office building which contains 20 lights, 10 ACs, and one dishwasher. Three scenarios have been designed to focus on different functionalities. The outcomes of the paper include proof of the performance of the optimization algorithm and how such a system can effectively minimize electricity consumption by implementing demand response programs and using them in smart grid contexts.

## 1. Introduction

The work in this paper has been done in the sequence of the work presented in [1]. Sometimes unimportant actions during the day can impact the environment and, in the end, affect the survival of the earth [2]. That has been the motivation for automation in buildings in order to prevent the loss of energy as much as possible [3]. Some strategic services such as demand response (DR) programs organize users’ consumption patterns as a generic and systematic program, in which electricity price variations or technical issues with consideration of the interests of consumers and producers are used to activate changes in the consumption patterns. According to the Federal Energy Regulatory Commission, Washington, D.C., USA (FERC) [4], a DR program is defined as “changes in electric use by demand-side resources from their normal consumption patterns in response to changes in the price of electricity, or to incentive payments designed to induce lower electricity use at times of high wholesale market prices or when system reliability is jeopardized”. The concept of DR programs is widely discussed at the scientific and research levels [5]. In most of the cases, DR programs have only been focused on consumption, which brings flexibility to the grid by paying incentives to the electricity consumers [6]. In fact, buildings are responsible for 40% of the world’s energy consumption which is increasing every day [7].

We have been working on optimizing the energy consumption in buildings while respecting the user’s preferences, specifically, considering the flexibility of lights and air conditioners [1]. However, previous methodologies, in line with the literature in the field, have not been adequately formalized. They have not been tested in a way that they could be used in a real building, in real time, with consideration of the dynamic changes of users, such as in an office building with consideration of both temperature and users’ preference constraints, and therefore integrated into a building energy management system with consideration of both the building manager and the users’ preferences. The motivation and objectives of this research are based on this referred lack of such a complete methodology and respective validation in a real building. As a hypothesis, we have developed and implemented the proposed methodology, which integrates the following features:

- multiperiod optimization to minimize the power consumption of an office building;
- updating of the optimization results according to the most recently updated users’ preferences;
- schedule of a dishwasher (DW), as a mean of consumption shifting, where the complete washing cycle is integrated into the energy management of several consecutive periods;
- constraining of the optimization problem by considering the visual comfort and thermal comfort of the users, with simple rules;
- adequate running of the optimization algorithm in successive periods, namely, a feature where the optimization runs every time that the consumer settings are changed;
- decision on when and how frequently the optimization algorithm is run;
- minimization of the temperature difference between target and actual temperature as part of the objective function, as a measure of comfort.

The following objectives have been successfully accomplished:

- the algorithm is implemented and integrated in an agent-based Supervisory Control and Data Acquisition (SCADA) system installed in a real office building, where all the parameters, such as the consumption of devices and the total consumption, are monitored through this SCADA system;
- three scenarios have been implemented and the results are collected in order to prove the adequacy of the proposed methodology;
- a sensitivity analysis was conducted regarding the role of parameters in power consumption and user comfort;
- the building energy manager has defined a set of parameters in order to specify the moments when the optimization is run.

The innovative scientific contribution of this paper is the integration of linear programming optimization in a real building energy management system, minimizing the discomfort of users in each room, using both temperature and power consumption parameters, deciding where to allocate the operation of a dishwasher in its complete cycle, and providing different means for users and for building energy managers to set the optimal bounds of operation for the building regarding its electrical appliances. The comfort approach uses the dynamic energy balance equation from [8] to relate the indoor and the outdoor temperatures in two successive time steps, and several equations that bound the energy usage by each appliance as defined by the user. Other approaches can be found in the literature; however, this approach represents a good compromise between complexity and accuracy.

After Section 1, the background literature is presented in Section 2. The optimization algorithm and the implemented methodology are explained in Section 3. A case study is demonstrated in Section 4 in order to validate the performance of the proposed optimization algorithm. Section 5 illustrates the obtained results of the proposed case study in Section 4. Finally, Section 6 describes the main conclusions of the work.

## 2. Background Literature

Among all types of buildings, office buildings are considered to be the more flexible option for implementing DR programs, because usually they have significant energy consumption, and also in some cases are better equipped for automation infrastructure than residential houses [9]. Recently, a main concern in energy consumption minimization topics is user comfort [8] and keeping a balance between energy consumption minimization and user preference needs that are formulated with adequate constraints in order to observe optimization purposes and user easement at the same time [10]. Mostly, in office buildings, more attention is paid to air conditioners (ACs) [11,12], while according to [13], 29% of the total energy consumption in office buildings occurs due to lighting. The lights in offices are considered as flexible loads if these are controllable by existing equipment [14].

A Supervisory Control and Data Acquisition (SCADA) system plays a key role in DR implementation since it offers various advantages in order to have automatic load control in different types of buildings [15]. For example, the SCADA system can control the lights in a building via a Digital Addressable Lighting Interface (DALI) [16], which has been implemented to support the results of this paper.

In the field of energy consumption, optimizations considering user comfort, various models, and control methods have been developed to reduce energy consumption, energy cost, and thermal discomfort. In [8], an automated DR implemented in a home energy management system is proposed to optimize the consumption of distinct types of domestic appliances in response to dynamic electricity prices. The main purpose of [8] is to reduce the consumer’s electricity bill, while increasing their comfort. For thermal appliances, the controller should find the corresponding power consumption at each time slot to preserve the preferred temperature of the user. In [17], a simplified preconditioning scenario was applied to control the annual energy cost and thermal comfort. The daily discomfort score was considered to evaluate the HVAC optimization, because indoor thermal comfort is related to a user’s workplace health and productivity, the discomfort score based on the adaptive comfort standard, AHSRAE 55, and the optimum comfort indoor temperature based on the outdoor temperature. The main goal of the researchers in [18] was to achieve a comfortable and energy-efficient built environment. In this context, a multiobjective optimization framework based on a genetic algorithm was proposed to achieve significant potential for HVAC energy savings with consideration of user thermal comfort. A multiobjective optimization algorithm was proposed in [9] to improve economic efficiency and maintain user comfort at the same time. This optimization algorithm is based on building integrated PV, noncontrollable loads, and outdoor temperature. In [19], a solution was proposed that considered a proper AC operating temperature based on probabilistic power consumption considering high-dimensional stochastic variables. A refined temperature control scheme was calculated to make the AC operate more efficiently. The main purpose of [20] was to minimize the electricity cost based on dynamic electricity price and minimize the thermal discomfort associated with a HVAC system. These minimizations were achieved by considering the uncertainties of electricity price, outdoor temperature, the most comfortable temperature level, and external thermal disturbances. In [21], the thermal behavior of related zones was modeled using an artificial neural network approach to predict the temperature of the next time slot, the energy demand for cooling the zone in the next time slot, and the zone occupancy. An energy reduction strategy was applied based on thermal feedback and comfort margin. In [22], an optimization problem to minimize the power fluctuation and maximize user comfort based on ASHRAE standard 55 was presented. For this purpose, a graph-based multiobjective optimal scheduling approach was proposed to manage high consumption devices such as ACs, with the goal of obtaining a smoother load profile and maximize users’ thermal comfortability.

In [13], the authors proposed a smart lighting control based on the internal mode controller of an artificial neural network which maintained occupant’s preferences while natural light was also used. In [15], a real model of an optimization-based SCADA model was presented, which focused on optimizing the energy usage of lights and AC devices of an office building in order to participate in DR events. Their results illustrated the importance of a SCADA system for DR programs, and how the demand side users are able to participate in DR programs while their preferences are not affected. In [23], a monitoring and controlling system was developed for lighting fixtures of different office spaces. The proposed work focused on the energy consumption of already installed devices, including lighting systems. The results of this work illustrated significant differences from the expected and real energy-saving results and, in the end, more reliable controlling and monitoring solutions were outlined.

The predicted mean value (PMV) model of thermal comfort, was created by Fanger et al. as an index that predicts the mean thermal sensation vote on a standard scale for a large group of persons for any given combination of thermal environmental variables, activity, and clothing levels [24]. The PMV index has been used in many studies to control the thermal comfort. In [25], a numerical simulation was made in a university building with 2072 occupants. The building was equipped with an HVAC system integrating a control physiology based on a PMV index. The PMV index was calculated as a function of the air temperature, air velocity, air relative humidity, mean radiant temperature, clothing level, and activity level to control the thermal behavior.

Considering the research in the literature, and in line with the research in this paper, it is noteable that several studies address optimized control of ACs and lights and the proposed models evaluate the users’ comfort in AC systems considering the distance to the desired temperature. Those works generally require a complex approach to validate the models with actual implementation in the real world. For example, the use of a PMV model requires nontrivial equipment and complex measurements which make its application impossible in practice for most of the AC and light control real-world applications. The input of a demand response signal to the optimization has also been disregarded. In fact, in order to validate the real implementation of optimization algorithms, adequate methodology must be provided, which is a goal in the proposed methodology of this work. The validation of the algorithm using real data and equipment is achieved. Moreover, with the proposed methodology, the building energy manager can define preferences in the optimization process, as building occupants are considered.

## 3. Methodology Description

**Figure 1:**
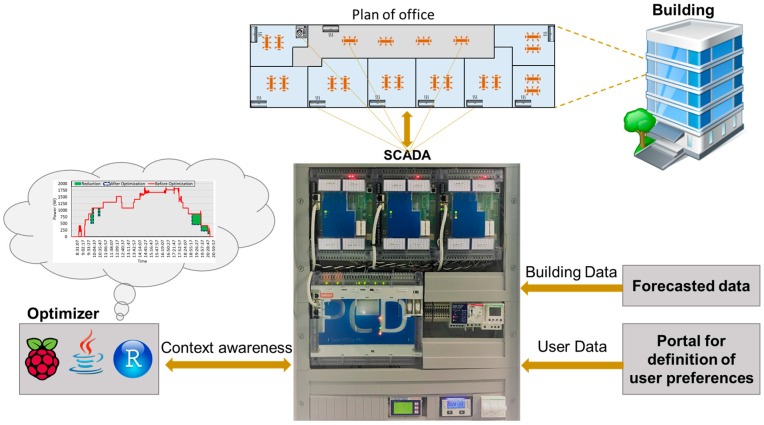
The overall architecture of the developed Supervisory Control and Data Acquisition (SCADA) system in an office building.

Section 3 describes the optimization algorithm implemented in the SCADA system in order to minimize the power consumption of the office building considering user preferences. The main purpose of the present methodology is to optimize the power consumption of the dishwasher (DW), lighting systems, and air conditioning (AC) system of the building. In fact, modification of user consumption patterns by an external entity could reduce user satisfaction. Therefore, several constraints should be considered to maintain user comfort. Figure 1 illustrates the overall system architecture.

**Figure 2:**
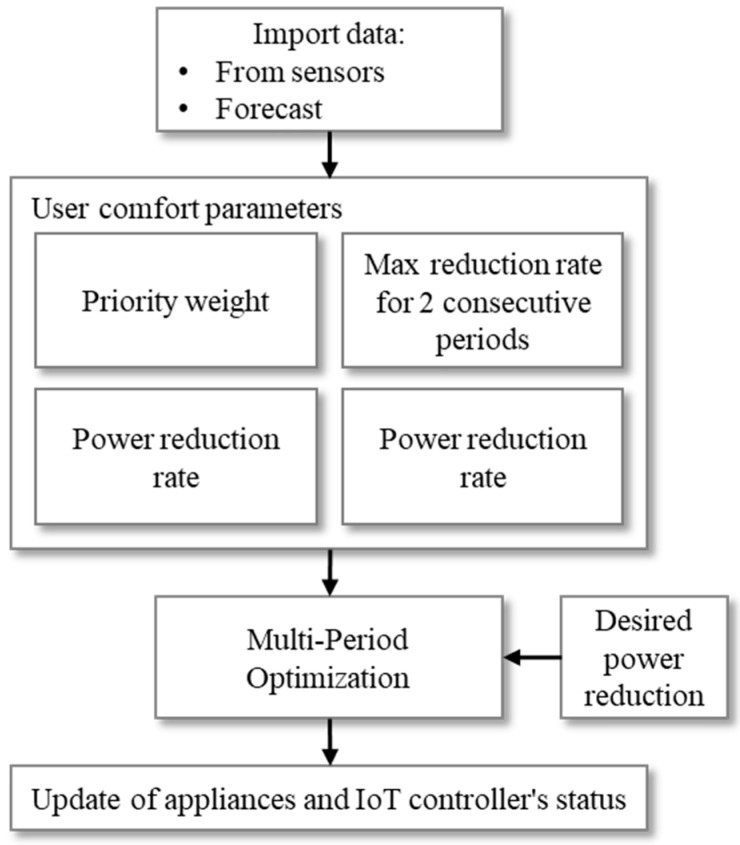
Flowchart of the proposed optimization methodology after each parameter update.

As shown in Figure 1, three types of appliances with different quantities are considered to achieve optimization purposes. Lighting intensity and ACs are employed to reduce the amount of power in different periods. A DW is employed for load shifting in peak hours to off-peak hours. Each of these operations should be performed in a specific framework. Figure 2 shows the process of the present optimization algorithm.

According to Figure 2, the algorithm starts by importing the input data such as consumption information of office buildings that have been provided by the SCADA system. The optimization algorithm presents a linear multiperiod optimization in which, in each period of the algorithm, different power reduction objectives are being addressed. There are priority numbers defined specifically for each distinct AC and light in order to determine the importance of each device in different periods. By this methodology, the devices compete to participate in power reduction based on their priority numbers. With respect to the definition of the priority, the lower numbers are the first participants in power reduction, while the high priority numbers are the most important and the most essential devices for the users. In the case that only one device is participating to achieve the optimization goals, such as the DW, the priority numbers show the importance of the time that a user employs that specific device.

Since the user preferences can change over time, the comfort parameters can be updated any time by users. After each parameter update, the algorithm runs with newly defined values and more recent updates for the energy consumption forecast.

According to the load shifting technique, the power consumption of the DW should be shifted from some periods to next or previous periods depending on the desired conditions, such as economic or technical reasons. In such appliances, the operation cycle is critical as it leads to several limitations in the operation scenarios, namely, when a DW starts to operate, where should the cycle be completed, and when can it not be interrupted before finishing the cycle. Therefore, the selection of the starting point for the DW load shifting in the optimization algorithm requires careful consideration. Accordingly, priority parameter figures have been dedicated to each period to determine the capability of each period to receive the shifted load.

**Figure 3:**
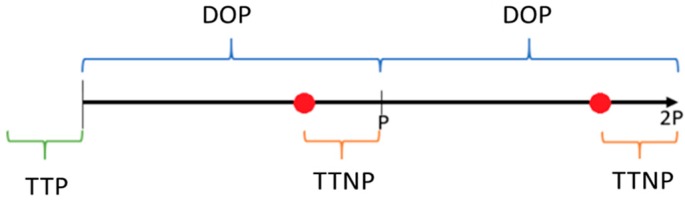
Timeline of the algorithm for processing data and starting point.

The present algorithm can consider several periods with various durations. The obtained results of each period are related to other periods, and the input data and parameters can be changed among all periods. In order to have a vision of the real implementation of the algorithm the TTP (required time to process the data), DOP (duration of the period), and TTNP (required time to start the next period of initial input data) have been considered as variables that can vary based on different utilizations. Figure 3 presents the timeline of the algorithm implementation.

As shown in Figure 3, the TTP is needed to import the initial data and input data of the first period to start the algorithm. It is also visible that the time required to obtain the results of the first period should be considered as the DOP-TTNP, while the TTNP is considered to import the required data and process the next period.

Regarding consideration of the user convenience, comfort parameters have been defined to limit the overall power reduction of each individual device and also limit the power reduction in consecutive periods. After adjusting all required parameters, the algorithm executes to achieve the desired power reduction and shift the DW load as needed, by considering thermal parameters and restriction parameters.

Equation (1) presents the objective function of the optimization algorithm.

(1) $$\begin{array}{ll} Minimize\;Objective & Function\\ & =\sum_{t=1}^{T}[\sum_{l=1}^{L}{W\_L}_{\left(l,t\right)}\times {P\_L}_{\left(l,t\right)}\\ & +\sum_{a=1}^{A}{W\_AC}_{\left(a,t\right)}\times {P\_AC}_{\left(a,t\right)}\\ & +\sum_{d=1}^{D}{W\_DW}_{\left(d,t\right)}\times {DW\_Bin}_{\left(d,t\right)}+\sum_{a=1}^{A}\frac{{T\_in}_{\left(a,t\right)}-{T\_set}_{\left(a,t\right)}}{{T\_in}_{\left(a,t\right)}}]\\ & +\sum_{st=1}^{ST}{W\_DW\_sh}_{\left(st\right)}\times {DW\_Bin\_sh}_{\left(st\right)} \end{array}$$

As can be seen in Equation (1), the consumption levels of the lights and ACs are considered as integer variables. The normal and shifted modes of the DW are considered as binary variables to represent the participation of the DW in optimization. *DW_Bin_sh* stands for shifting the complete operation cycle of the DW load between one to *ST* and *T_in* indicates the indoor temperature of each office in each period that the algorithm should select the respective *T_in* based on following constraints. *T_set* shows the set temperature by the user that can be different in each period of a day.

Since all the periods are not capable of receiving the shifted load, *W_DW_sh* specifies the best time to start the operation. Suppose that a complete operation cycle of the DW is equal to N periods, therefore, *W_DW_sh* in periods from *ST_N* to *ST* should be adjusted so that the shifting is not valid in the referred periods.

Regarding the constraints, Equation (2) represents the amount of required power reduction and load balance in each distinct period. According to Equation (2), the reduced power from lights, ACs, and the dishwasher can be considered as the total power reduction in each period. It should be mentioned that, since the DW is a discrete device, the curtailed power from the DW is equal to the actual consumption of the DW. However, the shifted power of the DW should be considered as the power consumption. Therefore, the power reduction in lights and ACs should compensate for the consumption of shifted load to achieve the demand of power reduction.

(2) $$\begin{array}{ll} \overset{L}{\underset{l=1}{\sum }}P\_L_{\left(l,t\right)}\;+\;\overset{A}{\underset{a=1}{\sum }} & P\_AC_{\left(a,t\right)}\\ & +\;\overset{D}{\underset{d=1}{\sum }}P\_DW\_act_{\left(d,t\right)}\times DW\_Bin_{\left(d,t\right)}\\ & -\;\overset{ST}{\underset{st=1}{\sum }}P\_DW\_sh_{\left(st,t\right)}\;\times \;DW\_Bin\_sh_{\left(st\right)}=RR\;;\;\forall \;1\leq t\;\leq T \end{array}$$

Equation (3) guarantees that the DW can be shifted only once in periods between one to *ST*. This means the algorithm is obligated to find a particular time suitable for the starting point of the DW.

(3) $$\overset{ST}{\underset{st=1}{\sum }}DW\_Bin\_sh_{\left(st\right)}\;=\;1$$

In Equation (4), it is specified that the shifted power of the DW should be exactly equal to the consumption of the DW in an actual situation. According to the input data, the shifted consumption of the DW should not be less or more than the consumption of the DW in a normal situation.

(4) $$\overset{T}{\underset{t=1}{\sum }}\overset{ST}{\underset{st=1}{\sum }}P\_DW\_sh_{\left(st,t\right)}\;\;\times \;\;DW\_Bin\_sh_{\left(st\right)}\;=\;\overset{T}{\underset{t=1}{\sum }}\overset{D}{\underset{d=1}{\sum }}\;P\_DW\_act_{\left(d,t\right)}\;\times \;DW\_Bin_{\left(d,t\right)}$$

In addition, the consumption optimization of lights and ACs can disrupt the visual and thermal comfort of the users, respectively. To overcome this issue, several constraints have been defined to limit the exorbitant power reduction from referred devices. For ACs, the algorithm should find the corresponding power consumption at each period to preserve the set temperature by users. Equation (5) shows the relation of indoor and outdoor temperature in two successive periods [8].

(5) $$\begin{array}{c} T\_in_{\left(a,t\right)}\;=\;\left(inertia\;\times \;T_{in}{}_{\left(a,t-1\right)}\right)\;+\;\left(1-inertia\right)\;\times \;(T_{out}{}_{\left(a,t-1\right)}-(\frac{C}{TC}\;\times \;(P_{AC_{act}}{}_{\left(a,t-1\right)}-\\ P_{AC}{}_{\left(a,t-1\right)})));\;\forall \;1\leq a\;\leq A;\forall \;2\leq t\;\leq T;\forall \;T_{in}{}_{\left(a,1\right)}\;=\;K \end{array}$$

In Equations (6) and (7), the power reduction of the lights and ACs has been restricted by defining *PRR_L* and *PRR_AC,* respectively.

(6) $$\overset{T}{\underset{t=1}{\sum }}P\_L_{\left(l,t\right)}\;\leq \;\overset{T}{\underset{t=1}{\sum }}PRR\_L_{\left(l,t\right)}\;\times \;P\_L\_Nom_{\left(l,t\right)};\;\forall \;1\;\leq l\;\leq L\;$$

(7) $$\overset{T}{\underset{t=1}{\sum }}P\_AC_{\left(a,t\right)}\;\leq \;\overset{T}{\underset{t=1}{\sum }}PRR\_AC_{\left(a,t\right)}\;\times \;P\_AC\_Nom_{\left(a,t\right)};\;\forall \;1\;\leq \;a\;\leq \;A$$

*PRR_L* and *PRR_AC* are the maximum allowed power reduction for each device in different periods. When *PRR_L* and *PRR_AC* are equal to one, it means the device is available to turn off. Additionally, Equations (8) and (9) present the limitation of power reduction for lights and ACs, respectively, based on specific hours of the day.

(8) $$\begin{array}{c} \underset{t}{\sum }P\_L_{\left(l,t\right)}\;\leq \;\underset{t}{\sum }\overset{H}{\underset{h=1}{\sum }}\left(Max\_L\_h_{\left(l,h\right)}\times P\_L\_Nom_{\left(l,t\right)}\;\right);\;\forall \;1\;\leq l\;\leq \;L;\\ \forall \;t\;=\;1-4;\;5-8;\;9-12;\;13-16;\;17-20;\;21-24;25-28;29-32;33-36;37\\ -40;41-44;45-48;49-T; \end{array}$$

(9) $$\begin{array}{c} \underset{t}{\sum }P\_AC_{\left(a,t\right)}\;\leq \;\underset{t}{\sum }\overset{H}{\underset{h=1}{\sum }}\left(Max\_AC\_h_{\left(a,h\right)}\times P\_AC\_Nom_{\left(a,t\right)}\;\right);\;\forall \;1\leq a\;\leq A;\;\\ \forall \;t\;=\;1-4;\;5-8;\;9-12;\;13-16;\;17-20;\;21-24;25-28;29-32;33-36;37\\ -40;41-44;45-48;49-T; \end{array}$$

Equations (8) and (9) are open to being adjusted based on users’ timetables and preferences. In the same equations, *Max_L_h* is the special state of *PRR_L*, and *Max_AC_h* is the appearance of *PRR_AC*. It should be noted that 1 h is divided into four periods in Equations (8) and (9).

Taking advantage of natural light in each room can be considered in this optimization. In fact, the amount of natural light depends on the architecture of each room, as well as geographical location of the room. Thus, Equation (10) sets the maximum allowed power reduction of each light based on the architecture and geographical conditions of the room with a defining *Max_L_R* parameter.

(10) $$\begin{array}{ll} \underset{l}{\sum }P\_L_{\left(l,t\right)}\;\leq \;\underset{l}{\sum } & Max\_L\_R_{\left(r,t\right)}\;\times \;P\_L\_Nom_{\left(l,t\right)}\;;\;\forall \;1\;\leq r\;\leq R\;;\;\forall \;1\;\leq t\;\leq T;\;\forall \;l\;\\ & =\;1-2;\;3-4;\;5-6;\ldots ;\;17-18;\;19-20;\;21-L \end{array}$$

Focusing on the user comfort, the power reduction in consecutive periods for a certain device can be annoying for the user because the user could feel light reduction or thermal inconvenience for a prolonged period. Therefore, Equations (11) and (12) are assigned to prevent power reduction os certain lights and ACs, respectively, in consecutive periods.

(11) $$P\_L_{\left(l,t\right)}\;+\;P\_L_{\left(l,\;t-1\right)}\;\leq \;MaxRed_{L}*P_{L_{Nom}}{}_{\left(l,t\right)}\;;\;\forall \;1\;\leq l\;\leq \;L;\;\forall \;2\;\leq \;t\;\leq T$$

(12) $$P\_AC_{\left(a,t\right)}\;+\;P\_AC_{\left(a,\;t-1\right)}\;\leq \;MaxRed_{AC}*\;P_{AC_{Nom}}{}_{\left(a,t\right)};\;\forall \;1\;\leq a\;\leq A;\;\forall \;2\;\leq t\;\leq T$$

As can be seen in Equation (11), the power reduction of each light in two consecutive periods is limited by *MaxRed_L* and the power reduction in each AC has been restricted by *MaxRed_AC* for two consecutive periods.

## 4. Case Study

The main purpose of Section 4 is to present illustrative scenarios to apply the proposed the methodology. The scenarios validate the methodology assumptions and detail the respective implementation. Three different scenarios are tested considering working hours of 20 days in an office building. The building, in this case study, is located on the Instituto Superior de Engenharia do Porto (ISEP) Campus, in Porto, Portugal and is equipped with the SCADA system for controlling and monitoring several environmental parameters, as well as energy consumption and production. In fact, the building is able to manage electricity consumption and also to record the data in a database. There are several energy meters in the building that monitor the real-time consumption of lighting systems, sockets, and air conditioners. Therefore, in this case study, the profiles of devices are real consumption data of the building that have been acquired from the SCADA system.

**Figure 4:**
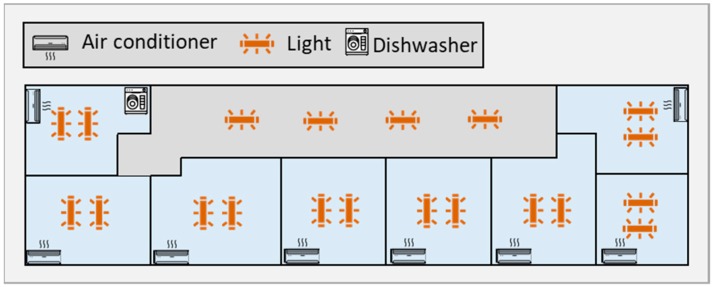
The plan of the building and controllable devices.

All comfort parameters used in Section 4 are adapted from several real experiments. The temperature variation in offices in the sequence of power consumption reduction has been investigated in many real studies to determine the thermal comfort limit. In addition, the visual comfort bounds of the users have been provided due to many actual tests with user interaction. Figure 4 illustrates the plan of the building and the controllable devices in each office room.

The lighting system of the building contains 20 fluorescent lights which are fully controllable via the Digital Addressable Lighting Interface (DALI) system. There are 10 controllable ACs that are located in each distinct area of the building, and one DW in the kitchen.

**Figure 5:**
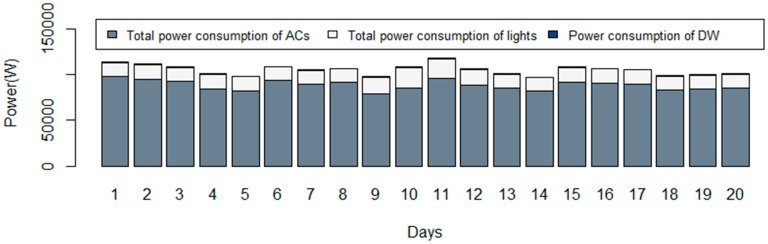
The power consumption of the controllable devices in each day.

In this case study, three different scenarios are shown to compare the results obtained for the different purposes of the algorithm. The amount of power reduction and cost reduction are compared in all scenarios with the base situation. Figure 5 shows the consumption of the DW, the lighting systems, and ACs of the building in 20 working days. It should be mentioned that the algorithm focuses on the working hours of the office building from 8:00 to 20:00. The 12 working hours have been divided into 48 periods with DOP equal to 15 min.

As shown in Figure 5, the ACs are responsible for a large part of the energy consumption in the building. Although, lighting systems and the DW can play effective roles in energy optimization.

Three different scenarios have been designed based on the same algorithm with various properties. All scenarios pursue the energy optimization goal while they have their particular purposes. It should be mentioned that the initial power consumption, the amount of power reduction, and the priority of devices are the same in all six scenarios and they focus on 20 days of input data. Table 1 presents the applied objective function (OF), which is classified as OF (1) and OF (2). OF (1) is applied to minimize the power consumption of devices without consideration of thermal comfort of users in the equation, however, OF (2) is the complete form of objective function in (1), which aims to minimize the power consumption of devices and discomfort of the users. Equations (13) and (14) show the OF (1) and OF (2), respectively.

(13) $$\begin{array}{ll} OF(1)=\sum_{t=1}^{T}[ & \sum_{l=1}^{L}{W_{L}}_{\left(l,t\right)}\times {P_{L}}_{\left(l,t\right)}\\ & +\sum_{a=1}^{A}{W_{AC}}_{\left(a,t\right)}\times {P_{AC}}_{\left(a,t\right)}+\sum_{d=1}^{D}{W_{DW}}_{\left(d,t\right)}\times {{DW}_{Bin}}_{\left(d,t\right)}]\\ & +\sum_{st=1}^{ST}{W\_DW\_sh}_{\left(st\right)}\times {DW\_Bin\_sh}_{\left(st\right)} \end{array}$$

(14) $$\begin{array}{ll} OF(2)=\sum_{t=1}^{T}[ & \sum_{l=1}^{L}{W\_L}_{\left(l,t\right)}\times {P\_L}_{\left(l,t\right)}\\ & +\sum_{a=1}^{A}{W\_AC}_{\left(a,t\right)}\times {P\_AC}_{\left(a,t\right)}\\ & +\sum_{d=1}^{D}{W\_DW}_{\left(d,t\right)}\times {DW\_Bin}_{\left(d,t\right)}+\sum_{a=1}^{A}\frac{{T\_in}_{\left(a,t\right)}-{T\_set}_{\left(a,t\right)}}{{T\_in}_{\left(a,t\right)}}]\\ & +\sum_{st=1}^{ST}{W\_DW\_sh}_{\left(st\right)}\times {DW\_Bin\_sh}_{\left(st\right)} \end{array}$$

Table 1 also shows the applied comfort constraints in each scenario, which is classified as two categories of, first, (I) and, secondly, (II). Comfort constraints category (I) indicates the consideration of constraints (5) for controlling the power reduction of lights and ACs, respectively, to preserve the indoor temperature between user preferred temperature settings. Comfort constraints category (II) considers constraints (6), and (7) for restricting the power reduction of lights and ACs, respectively. It also considers constraints (8), and (9) for applying restrictions in specific periods and (10) is defined to limit the power reduction of the lights in specific places based on user preferences. In the last stage, constraints (11), and (12) are dedicated for preventing power reduction in consecutive periods in certain devices.

### 4.1. Scenario A

Scenario A is the version of the algorithm which aims to reduce the demand for power reduction by considering user comfort constraints category (II) and shifting the DW consumption. Ten ACs, 20 lights, and one DW have been considered in this scenario. Comfort constraints should distribute the power reduction among all devices based on restrictions. Shifting the power consumption from some periods to other periods can be interpreted that the power should be curtailed in some periods, but this curtailment should be compensated for in other periods as power consumption. The challenging point for the algorithm is the continuouss operation cycle of the DW and selecting the suitable periods to shift the complete operating cycle. Since the operation cannot be interrupted, the algorithm searches for the best starting point. In this scenario, priority of each period determines the starting period of shifting. This priority can reflect electricity cost, technical issue, critical periods, and user decisions.

### 4.2. Scenario B

Scenario B is considered to survey the impact of comfort constraints category (I) for providing thermal comfort of the users without considering comfort constraints category (II). Ten ACs, 20 lights, and one DW have been considered in this scenario for optimizing the energy consumption by reducing and shifting the power. Comfort constraints category (I) has an impact on power consumption of the ACs by preserving the desired temperature of the user based on outdoor temperature and set temperature. It should be mentioned, in this study, that the inertia factor is equal to 0.98, the thermal conductivity *TC* = 0.35 kW/°C, and the coefficient of performance C = 5.

### 4.3. Scenario C

Scenario C is the complete version of the algorithm that addresses the impact of considering comfort constraints categories (I) and (II) on the behavior of 10 ACs, 20 lights, and one DW in optimization. The demand for total power reduction in each period is equal to scenario A and B, but the comfort constraints should distribute the power reduction among all devices based on defined thermal comfort constraints and power restrictions constraints. It should be noted that load shifting is also considered in this scenario. In this scenario, comfort constraints category (II) is able to restrict the power reduction based on the value of the parameters and required reduction; however, comfort constraints category (I) aims to obtain the desired indoor temperature.

### 4.4. Scenario Data

As shown in Table 2, the parameters that are related to the comfort of the user maintain their base value in all scenarios in order to survey the impact of added constraints, however they can be decreased or increased based on different purposes.

**Figure 6:**
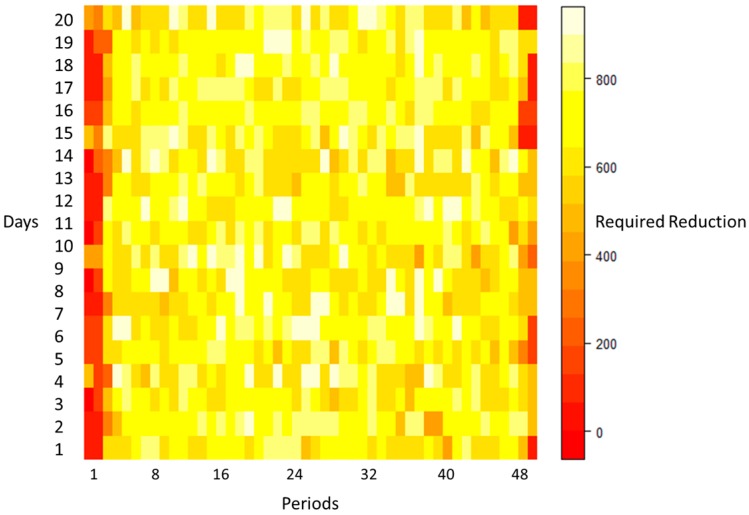
The demand for power reduction in each period for 20 days.

As shown in Figure 6, there are different demands for power reduction in each period of each day. These values are the minimum demand for power reduction; however, they can be increased in some periods to compensate the power consumption due to DW shifting.

**Figure 7:**
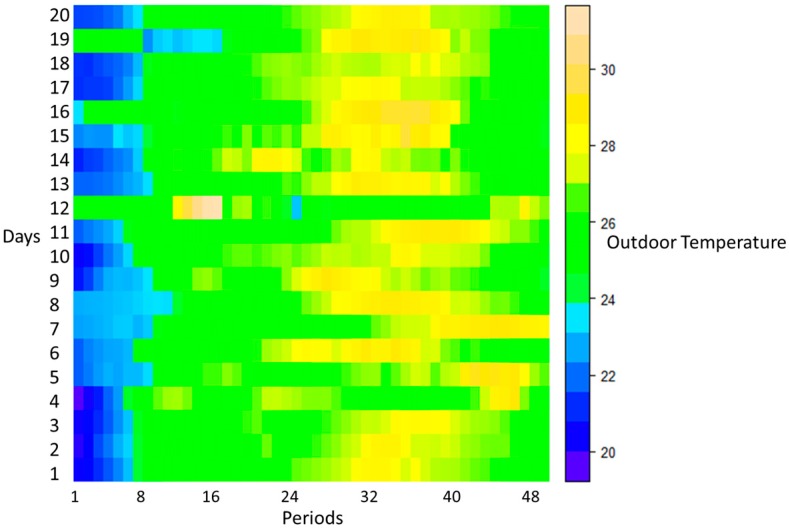
Outdoor temperature in each period for 20 days.

**Figure 8:**
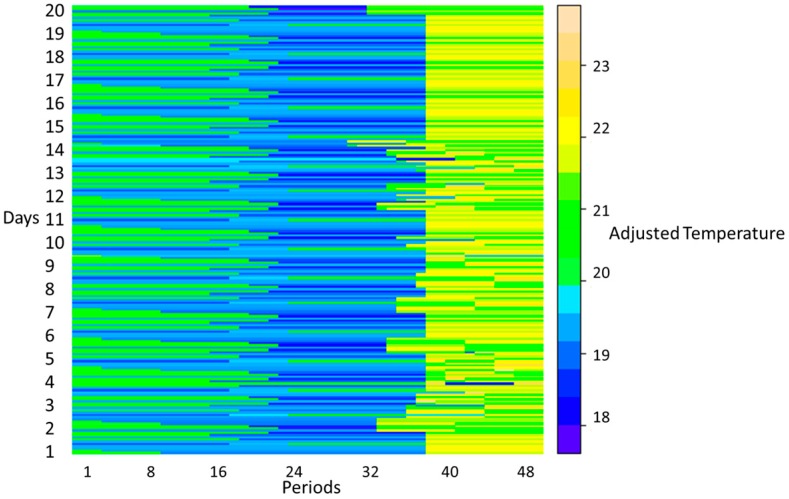
Adjusted temperature by users in each period for 20 days.

Figure 7 and Figure 8 show the outdoor temperature and the adjusted temperature by user, respectively, in each period of 20 days. It should be noted that Figure 8 shows the adjusted temperature by users in 20 days, however, each day includes 10 distinct AC with 10 different preferred temperature, and therefore Figure 8 shows 200 rows related to 20 days.

**Figure 9:**
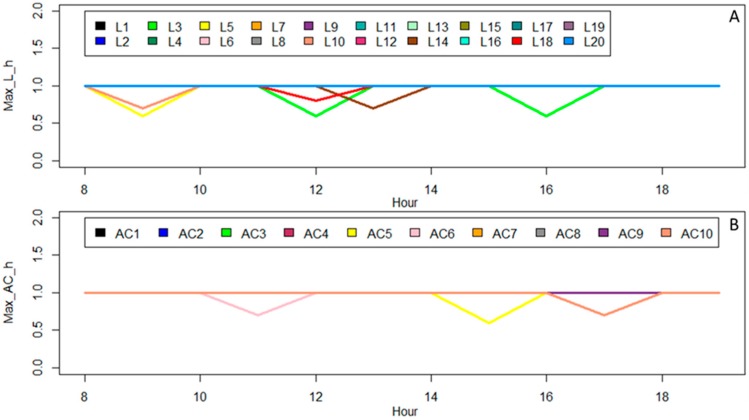
Coefficients regarding maximum allowed power reduction of lights and ACs. (**A**) amount of Max_L_h; (**B**) amount of Max_AC_h.

Figure 9A,B shows the amount of Max_L_h, and Max_AC_h, respectively, that are equal in all presented scenarios, but they can be modified based on users. According to the definitions of Max_L_h and Max_AC_h, they can limit the power reduction of devices in some particular hours, and therefore various amounts can be dedicated to them during a day.

Sometimes there are no special preferences for some devices and Max_L_h and Max_AC_h should be considered equal to one. Hence, some devices overlap in Figure 9A,B. In Section 4, all the considered scenarios for validating performance of the algorithm have been presented and explained. These scenarios are defined so that all the possibilities and features of the algorithm can be tested and validated.

## 5. Results

Section 5 presents the obtained results of the proposed scenarios to verify the impact of defined constraints on consumption reduction. The sensitivity analysis of comfort parameters is presented.

### 5.1. Scenario A

**Figure 10:**
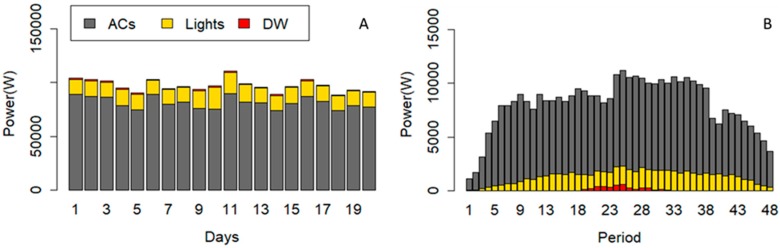
Power consumption of the devices after optimization in scenario A. (**A**) is correlated to power consumption of devices in each day, and (**B**) shows the average power consumption of devices in each period.

In this scenario, the required power reduction has been reduced from ACs and lights based on defined user comfort constraints (6) to (12) without considering thermal constraints. The power consumption of the DW is shifted from some periods to other periods. Figure 10 illustrates the power consumption of devices after running the optimization algorithm of Scenario A. Figure 10A is correlated to power consumption of devices in each day, and Figure 10B shows the average power consumption of devices in each period. The comparison of Figure 5 and Figure 10A indicates the reduced power from lights and ACs in scenario A. The total power consumption of the DW has not been changed in scenario A, while the operation cycle could be shifted. According to Figure 10B, the operation cycle of the DW has been placed between period 19 to 32.

**Figure 11:**
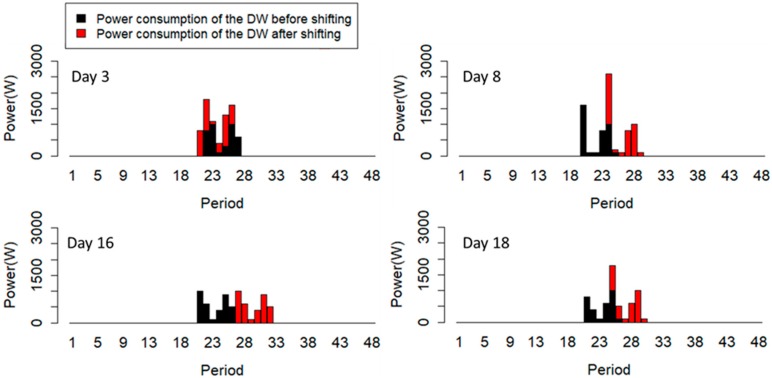
Load shifting of the DW on four selected days.

Four different consumption profiles of the DW can be seen in Figure 11. The implementation of load shifting is based on the weight of each period, and also capability of six successive periods to receive the complete operating cycle. For example, on Day #3, the algorithm decided to start the operation cycle one period before, based on the capability of the periods. The power consumptions of the DW on Day #8, Day #16, and Day #18 have been shifted to their first opportunity to shift, based on the importance weight of periods.

### 5.2. Scenario B

**Figure 12:**
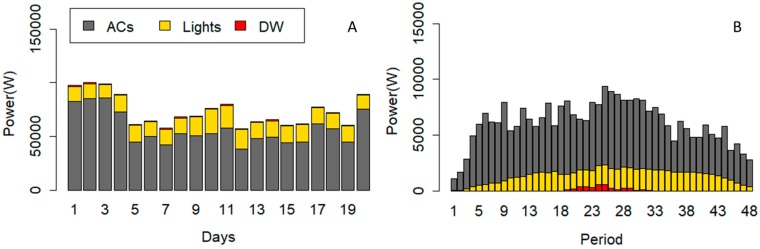
Power consumption of the devices after optimization in scenario B. (**A**) is correlated to power consumption of devices in each day, and (**B**) shows the average power consumption of devices in each period.

The obtained results of this scenario present the modification in consumption pattern of devices based on thermal constraints. It is clear that thermal constraint (5) is related to thermal comfort of the users and it is expected to have an effect on the power consumption of the ACs. Figure 12 shows the power consumption of devices after running the optimization algorithm of Scenario B. Figure 12A is correlated to power consumption of devices in each day, and Figure 12B shows the average power consumption of devices in each period. The comparison of Figure 5, Figure 10A, and Figure 12A shows the reduced power from the lights and ACs in scenario B. Similar to scenario A, the total power consumption of the DW has not been changed in scenario B, while the operation cycle could be shifted. According to Figure 12B, the operation cycle of the DW has been placed between period 19 to 32.

**Figure 13:**
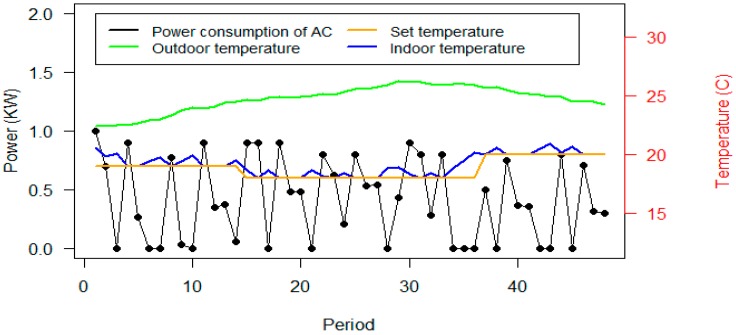
Power consumption of the AC and thermal parameters of Office #2, Day #13, in scenario B.

As shown in Figure 12, the power consumption of the ACs has been significantly reduced in scenario B. However, the power consumption of lights is increased. The required reduction of scenario A and B are equal, however constraint (2) justifies that the power consumption of the devices have been reduced more than the required reduction. According to constraint (5), it can be interpreted that these power consumptions achieved the set temperature by the user based on the proposed parameters. Figure 13 shows the consumption and thermal situation of Office #2 on Day #13.

According to Figure 13, the algorithm has obtained the indoor temperature close to the set temperature by user. As the outdoor temperature is increased, the power consumption of the ACs also increased. It can be concluded from Figure 12 and Figure 13 that the desired temperature has been achieved with more than the required reduction of the algorithm.

### 5.3. Scenario C

**Figure 14:**
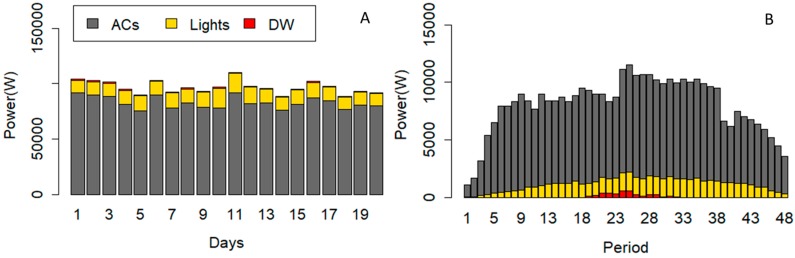
Power consumption of the devices after optimization in Scenario 6. (**A**) indicates the power consumption of devices on each day, and (**B**) shows the average power consumption of devices in each period.

The obtained results of this scenario present the impacts of all defined constrains on the consumption pattern of the devices. Thermal constraints affect the power consumption of the ACs, while the comfort constraints restrict ACs and lights. Figure 14 shows the power consumption of devices after running the optimization algorithm of Scenario C. Figure 14A indicates the power consumption of devices on each day, and Figure 14B shows the average power consumption of devices in each period. The comparisons of Figure 5, Figure 10A, Figure 12A, and Figure 14A show the reduced power from lights and ACs in scenario B. Similar to scenario A and B, the total power consumption of the DW has not been changed in scenario C, while the operation cycle could be shifted. According to Figure 14B, the operation cycle of the DW has been placed in the periods 19 to 32.

According to the comparison of Figure 10 and Figure 14, the power consumption of ACs have been increased in scenario C; however, the power consumption of the lights has been decreased.

**Figure 15:**
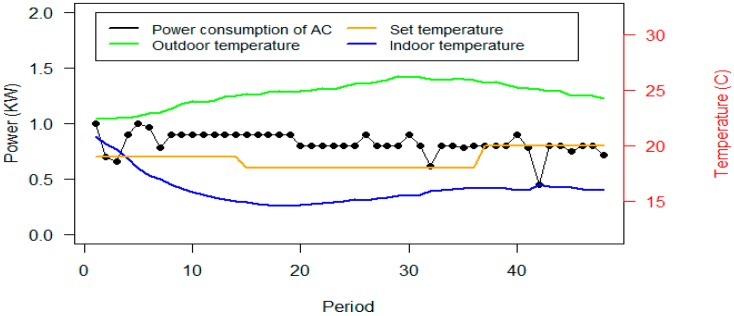
Power consumption of the AC and thermal parameters of Office #2, Day #13, scenario C.

From the comparison of scenarios B and C that consider thermal comfort constraints, it can be seen that the power consumption of the ACs in scenario C is more than the power consumption of the ACs in scenario B. Since all parameters as such required reduction, the outdoor temperature and the set temperature are equal in both scenarios, similar to scenario B. Office #2 on Day #13 has been selected to show the consumption and thermal situation. Figure 15 shows the consumption and thermal situation of Office #2 on Day #13.

As shown in Figure 15, the achieved indoor temperature is less than the set temperature by the user. The power consumption of the ACs in scenario C is higher than scenario B and it decreases the indoor temperature. It can be observed that the comfort restrictions do not let the algorithm reduce the power consumption of the ACs. Although scenario C could not achieve the desired temperature of the user, scenario B also denied the user the freedom to act on the required reduction.

**Figure 16:**
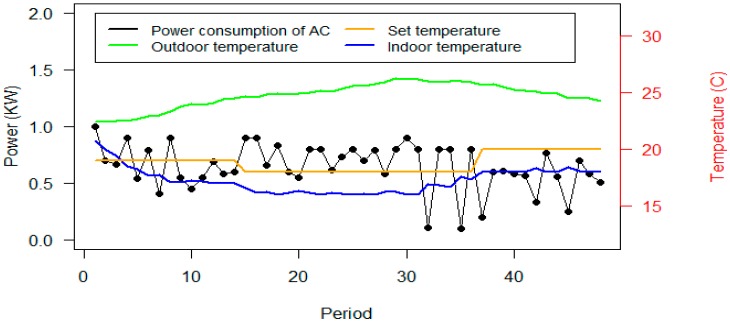
Power consumption of the ACs and thermal parameters of Office #2 on Day #13 in scenario C after 25% increment of comfort parameters.

According to the comparison of three scenarios, it can be interpreted that comfort parameters in constraints (6) to (12) require an overview in scenario C. In order to verify the impact of comfort constraints, all comfort parameters in constraints (6) to (12) have been increased by 25% of the authorized values in scenario C. Figure 16 presents the consumption and thermal situation of Office #2 on Day #13.

As shown in Figure 16, by increasing 25% of the comfort parameters defined in constraints (6) to (12), the indoor temperature is close to the set temperature by users. In addition, the consumption of the AC devices has been changed accordingly based on the applied modification.

### 5.4. Sensitivity Analysis of Power Consumption Regarding Constraints Bounds Variation

**Figure 17:**
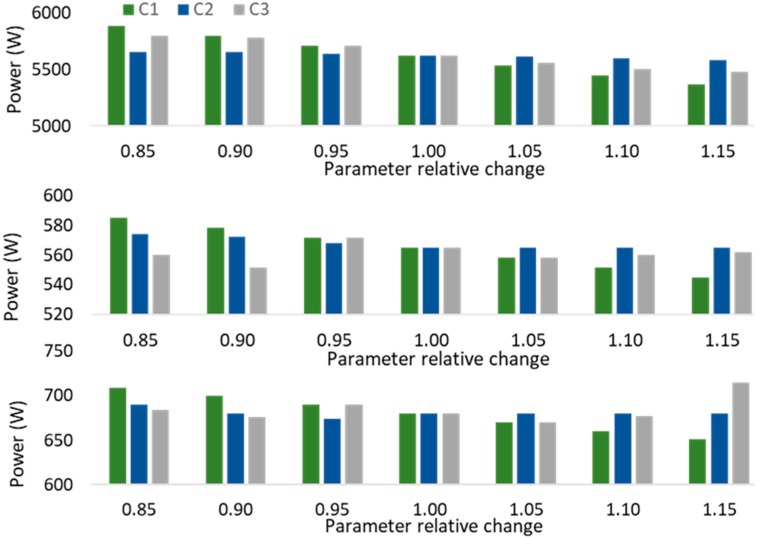
Consumption of devices in Room #5 regarding sensitivity analysis of constraint bounds.

According to Table 2 and Figure 9, the comfort parameters maintain constant values in comfort scenarios to address the impacts of different constraints. However, the impact of the parameters should be surveyed. For this purpose, scenario A has been considered as the base case, and Figure 12 presents the obtained results of the sensitivity analysis of power consumption regarding variations of parameters in Room #5. It should be mentioned that Room #5 contains L #5, L #6, and AC #5 and their related parameters are visible in Table 2, and Figure 9. Comfort 1 (C1) focuses on variations of constraints (6) and (7) while constraints (8) to (12) are equal to base values in scenario A. Comfort 2 surveys the variations of constraints (8), (9), and (10) while constraints (6), (7), (11), and (12) maintain their base values in scenario A. Comfort 3 (C3) considers the variation of constraints (11) and (12) whereas constraints (6) to (10) are in the base state of scenario A. In each class of C1, C2, or C3, the related parameters are multiplied by values which are presented on the *x*-axis of Figure 17.

As shown in Figure 17, AC #5, L #5, and L #6 are the existing devices in Room #5. The consumption pattern of AC #5 in C1 is clearly presenting that the consumption is decreasing while the PRR_AC base is increasing by its coefficients. It indicates that the increase in PRR_AC has decreased the limitations of power reduction. This also is visible for L #5 and L #6 in C1 due to variation of PRR_L. Regarding the definition of Max_L_h, Max_AC_h, and Max_L_R, they are designed to effect the power reduction of devices in some special periods or some special rooms. As shown in Figure 9, Max_L_h and Max_AC_h specified for L #5, L #6, and AC #5 is one h. The insignificant variations in the C2 bars indicate the impacts of the increment of parameters on consumption patterns only for one h.

The obtained results of the C3 study in AC #5 present that the increase in Max_Red_AC makes the AC free to reduce more power in two consecutive periods. However, this is different in the case of L #5 and L #6. They do not follow a recognizable pattern, because, in each variation of coefficients, the consumption patterns of all devices in all period are affected. In fact, constraints (11) and (12) are the constraints that affect all individual devices and all periods.

Since the power reduction is the same in all coefficient variations, any change in each device can cause changes in other devices. It is interpreted that, increasing the Max_Red_AC, and Max_Red_L causes an increase in power consumption of some devices, while other devices consume less power.

## 6. Conclusions

Demand response programs have great potential to improve buildings’ energy consumption optimization, bringing benefits to the building energy manager and occupants. Occupants comfort must be respected, and adequate automation should support such an intelligent energy management.

Research in the literature has focused on optimizing the air conditioning use according to the actual indoor temperature, in order to keep it close to the desired level, and in a set of offline scenarios. The optimization model proposed in this paper has been adequately developed in order to address the control of lights, ACs, and a washing machine, providing comfort modeling by keeping the desired temperature and by prioritizing the use of each appliance. Successful implementation in a real building has been achieved.

The results obtained in the three proposed scenarios show that the proposed methodology can optimize the energy consumption with an adequate modeling of the indoor temperature behavior. In Scenario B, the optimization was focused on the indoor temperature equations and it was possible to control the consumption of each appliance. In Scenario C, other constraints were considered in order to give the building energy manager and the users the opportunity to set bounds on the changes of the devices’ control actions. Easily, it was possible to identify the more relevant appliances in each period, and in each room, providing a more flexible contextual energy management. It is a major achievement of the proposed methodology because the users were able to specify their preferences regarding lights and ACs rather than consider only the indoor temperature setpoint. As shown in the results, as the bounds of the refereed equations are set to reduce the flexibility of each appliance, as a measure of comfort also, it was possible to analyze the indoor temperature behavior which was kept rather far from the desired temperature, as the users had other conflicting preferences. In fact, the provided objective function aims to reduce the difference between the actual and the desired temperature (in each individual room) but, as seen, if the users provide flexibility in the preference constraints, the optimization manages to reach the required demand reduction as a constraint, and therefore in some rooms the ACs provide a relatively bad temperature profile. The main point is that the model decides which are the most important ACs in each period for the global building target, respecting the individual users’ preferences. Such results are in fact very different from the results in the literature as those studies focused on keeping the indoor temperature without properly challenging obtaining a required reduction level.

As future work, sensitivity analyses should be done in order to study the definition of the importance of temperature difference minimization in the objective function.

## Figures and Tables

**Table 1:** Comparison of 3 scenarios based on their features.

Scenarios	Objective Function	Comfort Constraints I (5)	Comfort Constraints II (6), (7), (8), (9), (10), (11), (12)
A	OF (1)	-	√
B	OF (2)	√	-
C	OF (2)	√	√

**Table 2:** The parameter values for each scenario.

	Scenario A	Scenario B	Scenario C
DOP (In Minutes)	15	15	15
RR (2)	Figure 6	Figure 6	Figure 6
Inertia	0.98	0.98	0.98
TC	-	0.35	0.35
C	-	5	5
T_out	-	Figure 7	Figure 7
T_set	-	Figure 8	Figure 8
PRR_L (5)	0.4	-	0.4
PRR_AC (6)	0.6	-	0.6
Max_L_R (7)	Figure 9A	-	Figure 9A
Max_L_h (8)	Figure 9B	-	Figure 9B
Max_AC_h (9)	0.7	-	0.7
Max_Red_L (10)	0.6	-	0.6
Max_Red_AC (11)	0.46	-	0.46

## Nomenclature

Parameters

*TTP*	Required time to process the data
*DOP*	Duration of period
*TTNP*	Required time to start the next period
*W_L*	Importance weight of light
*P_L*	Power reduction of light
*W_AC*	Importance weight of air conditioner
*P_AC*	Power reduction of air conditioner
*W_DW*	Importance weight of the dishwasher
*DW_Bin*	Binary variable to indicate dishwasher state
*T_in*	Indoor temperature based on power consumption of AC
*T_set*	Adjusted temperature by user
*W_DW_sh*	Importance weight of each period for starting time of shifted power
*DW_Bin_sh*	Binary variable to indicate the state of shifted power of the dishwasher
*P_DW_act*	Actual power consumption of the dishwasher
*P_DW_sh*	The shifted power of the dishwasher
*RR*	Required power reduction
*inertia*	Inertia factor
*T_out*	Outdoor temperature
*C*	Coefficient of performance
*TC*	Thermal conductivity
*PRR_L*	The power reduction rate of light
*P_L_Nom*	Nominal power consumption of the light
*PRR_AC*	The power reduction rate of the air conditioner
*P_AC_Nom*	Nominal power consumption of the air conditioner
*Max_L_h*	Percentage of nominal power consumption that determines the maximum allowed power reduction of light in each h
*Max_AC_h*	Percentage of nominal power consumption that determines the maximum allowed power reduction of the air conditioner in each h
*Max_L_R*	Percentage of nominal power consumption that shows the maximum allowed power reduction of lights in each h
*MaxRed_L*	Maximum allowed power reduction from a certain light in two consecutive periods
*MaxRed_AC*	Maximum allowed power reduction from a certain air conditioner in two consecutive periods

Indexes

*L*	Number of lights
*A*	Number of air conditioners
*D*	Number of dishwashers
*T*	Number of periods
*ST*	Starting time for shifting
*H*	Hour
*R*	Number of rooms
